# Ultrahigh Power Factor in Thermoelectric System Nb_0.95_M_0.05_FeSb (M = Hf, Zr, and Ti)

**DOI:** 10.1002/advs.201800278

**Published:** 2018-05-02

**Authors:** Wuyang Ren, Hangtian Zhu, Qing Zhu, Udara Saparamadu, Ran He, Zihang Liu, Jun Mao, Chao Wang, Kornelius Nielsch, Zhiming Wang, Zhifeng Ren

**Affiliations:** ^1^ Institute of Fundamental and Frontier Sciences University of Electronic Science and Technology of China Chengdu 610054 China; ^2^ Department of Physics and Texas Center for Superconductivity University of Houston Houston TX 77204 USA; ^3^ Institute for Metallic Materials IFW‐Dresden Dresden 01069 Germany; ^4^ State Key Laboratory of Electronic Thin Films and Integrated Devices School of Microelectronics and Solid‐state Electronics University of Electronic Science and Technology of China Chengdu 611731 China

**Keywords:** half‐Heusler compounds, power generation, simultaneous optimization, thermoelectric materials

## Abstract

Conversion efficiency and output power are crucial parameters for thermoelectric power generation that highly rely on figure of merit *ZT* and power factor (PF), respectively. Therefore, the synergistic optimization of electrical and thermal properties is imperative instead of optimizing just *ZT* by thermal conductivity reduction or just PF by electron transport enhancement. Here, it is demonstrated that Nb_0.95_Hf_0.05_FeSb has not only ultrahigh PF over ≈100 µW cm^−1^ K^−2^ at room temperature but also the highest *ZT* in a material system Nb_0.95_M_0.05_FeSb (M = Hf, Zr, Ti). It is found that Hf dopant is capable to simultaneously supply carriers for mobility optimization and introduce atomic disorder for reducing lattice thermal conductivity. As a result, Nb_0.95_Hf_0.05_FeSb distinguishes itself from other outstanding NbFeSb‐based materials in both the PF and *ZT*. Additionally, a large output power density of ≈21.6 W cm^−2^ is achieved based on a single‐leg device under a temperature difference of ≈560 K, showing the realistic prospect of the ultrahigh PF for power generation.

## 1 Introduction

Thermoelectric materials are capable to directly convert heat to electricity, providing an effective route to recover waste heat.^1^, ^2^, ^3^, ^4^, ^5^ The conversion efficiency η is determined by Carnot limit and materials' property, figure of merit *ZT*, defined as *ZT* = (*S* ^2^σ/κ)*T*, where *S*, σ, κ, and *T* are the Seebeck coefficient, the electrical conductivity, the total thermal conductivity, and the absolute temperature, respectively, and the product (*S* ^2^σ) is called power factor (PF).^6^, ^7^, ^8^ A higher *ZT* will result in a higher η, therefore improvement of *ZT* has been the goal for the thermoelectric community.

One effective strategy for *ZT* improvement is to minimize the lattice thermal conductivity by scattering phonons off crystal defects, e.g., grain boundaries,^9^, ^10^, ^11^, ^12^, ^13^, ^14^ dislocations,^15^, ^16^, ^17^, ^18^ point defects,^19^, ^20^, ^21^, ^22^, ^23^, ^24^, ^25^ nanoprecipitates,^26^, ^27^, ^28^, ^29^, ^30^ etc. However, thermoelectric power generation demands not only high *ZT* but also high PF over a wide range of temperature, which directly determines output power density ω, another crucial parameter for power generation.^31^, ^32^, ^33^ Frustratingly, due to the intrinsic conflicts of the parameters, simultaneous optimization of electric and thermal properties is extremely difficult. Most of approaches to increase the phonon scattering also cause carrier scattering, resulting in reduced carrier mobility and decreased PF. Therefore, suppressing lattice thermal conductivity without affecting the carrier mobility and PF becomes a prime issue for realizing high power thermoelectric generators.^6^, ^34^, ^35^

Among the various high‐performance thermoelectric materials, half‐Heusler (HH) compounds, with a formula ABZ, where A can be an early transition metal or a rare‐earth element, B is a less electropositive transition metal, and Z is a main group element, are identified as promising materials for power generation due to their intrinsically high PF^36^, ^37^, ^38^, ^39^, ^40^ and good thermomechanical stability.^41^ But the large lattice thermal conductivity originating from their simple crystal structure and strong bonding impairs high *ZT* achievement,^42^ hence nanostructuring and isoelectronic alloying were employed for reducing the lattice thermal conductivity, but also detrimental to the carrier mobility. For instance, although strong phonon scattering contributes to p‐type ZrCoSb achieving benchmark *ZT* of ≈1 at 1100 K, the mobility is usually less than 10 cm^2^ V^−1^ s^−1^ at 300 K, thus the peak PF is only around 30 µW cm^−1^ K^−2^.^21^, ^43^, ^44^ It is worthwhile to note that ZrNiSn‐based n‐type HH compounds possess a relatively higher mobility (>20 cm^2^ V^−1^ s^−1^ at 300 K) with a peak PF of ≈50 µW cm^−1^ K^−2^ and benchmark *ZT* of ≈1 at 900–1100 K.^45^, ^46^, ^47^, ^48^, ^49^ Recently, the compound NbFeSb renewed the development of p‐type HH,^13^, ^33^, ^50^, ^51^, ^52^, ^53^, ^54^, ^55^ such as peak PF of ≈60 µW cm^−1^ K^−2^ at 700 K and *ZT* of ≈1.5 at 1200 K obtained in a heavy‐band p‐type NbFeSb.^51^ Nevertheless, high content of dopants (e.g., 40% Ti at Nb site^50^) or isoelectronic substitutions (e.g., 40% Ta at Nb site^55^) were mainly designed to scatter phonons, causing the mobility below 20 cm^2^ V^−1^ s^−1^ at 300 K, implying the possibility of achieving higher PF through improving mobility. Additionally, Rogl and co‐workers investigated the phase diagram and transport properties at low temperature of the Nb–Fe–Sb system,^54^ but the peak PFs are still similar with the values reported by Fu et al.^51^ (≈60 µW cm^−1^ K^−2^) which is due to the nonoptimized defect scattering and strong ionized impurity scattering from the relatively high content of dopants.

**Figure 1:**
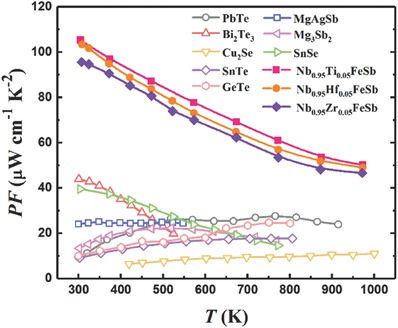
Comparison of temperature‐dependent PF among Nb_0.95_M_0.05_FeSb (M = Hf, Zr, Ti) and other high‐performance thermoelectric materials.^10^, ^12^, ^29^, ^33^, ^35^, ^56^, ^57^, ^58^, ^59^

In this work, we demonstrate a material system Nb_0.95_M_0.05_FeSb (M = Hf, Zr, Ti) with significantly enhanced PF to ≈100 µW cm^−1^ K^−2^ at room temperature. Such a substantial enhancement is mainly ascribed to the improved carrier mobility (>20 cm^2^ V^−1^ s^−1^ at 300 K). As a result, this p‐type NbFeSb system possesses more competitive PF than other high‐performance thermoelectric materials (e.g., lead telluride, bismuth telluride, etc.^10^, ^12^, ^29^, ^35^, ^56^, ^57^, ^58^, ^59^) over a wide temperature range, as shown in **Figure** **1**. Due to simultaneously optimize carrier mobility and suppress lattice thermal conductivity, Nb_0.95_Hf_0.05_FeSb is unique for the highest *ZT* of ≈0.9 at 973 K in this material system, but with the ultrahigh PF maintained. In comparison with another outstanding p‐type NbFeSb (*ZT* ≈1.5 at 1200 K),^51^ our Nb_0.95_Hf_0.05_FeSb shows a higher ω though the η is a little bit lower. Furthermore, a quite large output power density ω of ≈21.6 W cm^−2^ is achieved based on a single‐leg device under a temperature difference of ≈560 K, demonstrating the great potential of Nb_0.95_Hf_0.05_FeSb for power generation.

## 2 Results and Discussion

**Figure 2:**
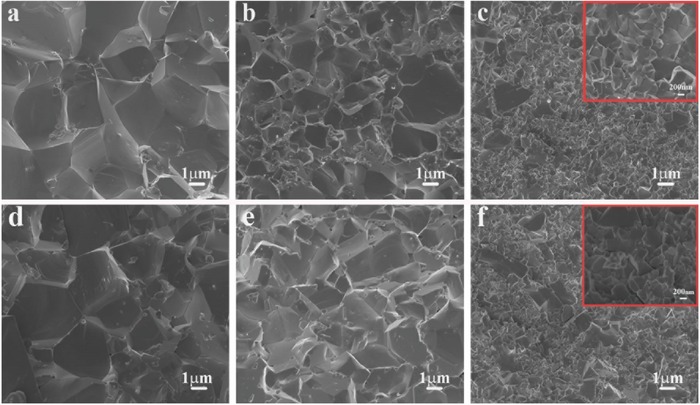
SEM images of a–c) Nb_0.95_Hf_0.05_FeSb and d–f) Nb_0.95_Zr_0.05_FeSb hot pressed at 1323, 1273, and 1173 K, respectively. The insets of (c) and (f) correspond to the samples hot pressed at 1173 K with high magnification for easier recognition of the grain size.

X‐ray diffraction (XRD) spectra of all samples are shown in Figure S1 (Supporting Information), where the diffraction peaks correspond well with MgAgAs‐type structure (space group $F\bar{4}3m$), indicating pure HH phase for all samples. **Figure** **2** shows the scanning electron microscope (SEM) images of Nb_0.95_Hf_0.05_FeSb and Nb_0.95_Zr_0.05_FeSb with hot pressing (HP) temperatures of 1173, 1273, and 1323 K, where the increased grain size from hundreds of nanometers to a few micrometers with HP temperature was clearly observed. In addition, lack of pores within the large areas indicates the uniformity of samples with high density (Figure S2, Supporting Information), which was further confirmed by the relative density of >98% presented in Table S1 (Supporting Information). The uniform element distribution in samples hot pressed at higher and lower temperature was observed, as shown in Figures S3 and S4 (Supporting Information).

**Figure 3:**
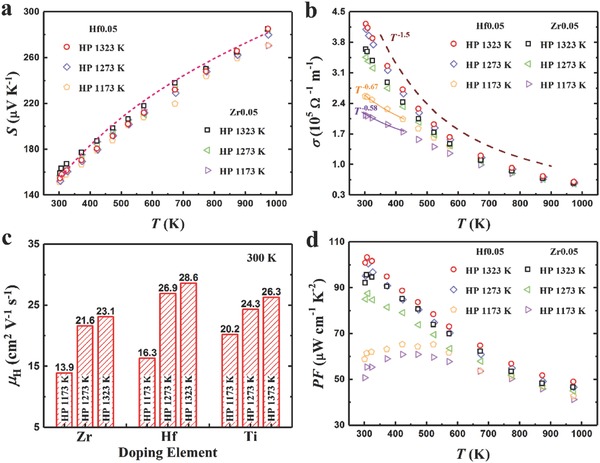
Temperature‐dependent electrical properties of Nb_0.95_Hf_0.05_FeSb and Nb_0.95_Zr_0.05_FeSb hot pressed at 1323, 1273, and 1173 K. a) Seebeck coefficient, b) electrical conductivity, c) Hall mobility at 300 K, together with the data of Ti doping,^33^ and d) power factor.

**Figure** **3** shows the temperature‐dependent electrical properties of Nb_0.95_Hf_0.05_FeSb and Nb_0.95_Zr_0.05_FeSb. In Figure 3a, the *S* curves are almost overlapped showing weak dependence on either HP temperature or doping element, which is mainly due to their similar doping concentrations and band structures. The estimated *S* based on the single parabolic band (SPB) model (shown by the dashed line) corresponds well with the experimental data. Here, the SPB model is described as

(1) $$S\;=\;+\frac{k_{B}}{e}\left[\frac{2F_{1}\left(\eta_{F}\right)}{F_{0}\left(\eta_{F}\right)}\;-\;\eta_{F}\right]$$

(2) $$F_{n}\;\left(\eta_{F}\right)=\int_{0}^{\infty }\frac{\chi^{n}}{1\;+\;\exp\left(\chi \;-\;\eta_{F}\right)}\;d\chi$$

where *k*_B_ is the Boltzmann constant, η_F_ is the reduced Fermi energy (η_F_ = *E*_F_/*k*_B_T), and *F_n_*(η_F_) is the Fermi integral of order *n*. As shown in Figure 3b, the σ decreases monotonically with temperature, which is the typical behavior of any degenerate or metal‐like semiconductor. Note that in contrast to the *S* barely affected by the HP temperature, a noticeable difference in the σ as function of the HP temperature was observed. For the samples hot pressed at higher temperatures (1273 and 1323 K), the behavior of σ follows the *T* ^−1.5^ law, suggesting acoustic‐phonon‐dominated carrier scattering. While, the σ of Hf‐ and Zr‐doped samples hot pressed at 1173 K exhibit *T* ^−0.67^ and *T* ^−0.58^ dependence at lower temperature range, respectively, which implies the mixed scattering mechanism being dominant.^60^, ^61^ Basically, due to both Hf and Zr are quite efficient dopants to supply carriers, the similar *n*_H_ was achieved (see Table S1 in the Supporting Information). Hence, the disparity in σ originated from the varying *µ*_H_, which is very sensitive to the scattering mechanism.^60^, ^61^, ^62^ Figure 3c shows a significant *µ*_H_ improvement in the samples hot pressed at higher temperatures regardless of doping element, for instance, the *µ*_H_ of Hf‐doped sample hot pressed at 1323 K shows ≈75% higher than that at 1173 K. Such a substantial improvement benefits from the special nature of NbFeSb with rapid diffusion and grain growth at high temperatures, resulting in less grain boundaries and probably few point defects. This was also confirmed by the different temperature exponents for the σ–*T* curves. Owing to the similar *S* and improved *µ*_H_, the PF is significantly enhanced via hot pressing at higher temperature, as shown in Figure 3d, where the peak PF of Hf‐doped sample hot pressed at 1323 K is ≈103 µW cm^−1^ K^−2^ at 308 K and that of Zr‐doped sample is ≈97 µW cm^−1^ K^−2^. In addition to the ultrahigh peak value, Nb_0.95_M_0.05_FeSb (M = Hf, Zr, Ti) system also showed remarkable enhancement in PF over a wide range of temperature in comparison with other high‐performance thermoelectric materials (see Figure 1), indicating the great potential for power generation over a wide temperature range. Furthermore, understanding the carrier scattering mechanism plays a crucial role in optimizing the electrical properties of thermoelectric materials, such as Mao et al. demonstrated a significant enhancement in the mobility and PF of n‐type Mg_3.2_Sb_1.5_Bi_0.49_Te_0.01_ by carefully tuning the hot pressing temperature to manipulate the variation of scattering mechanisms,^61^ while few studies focusing on the scattering mechanism in HH.^63^, ^64^ Therefore, further systematical investigation on the carrier scattering mechanism in the materials with ultrahigh PF is still necessary.

**Figure 4:**
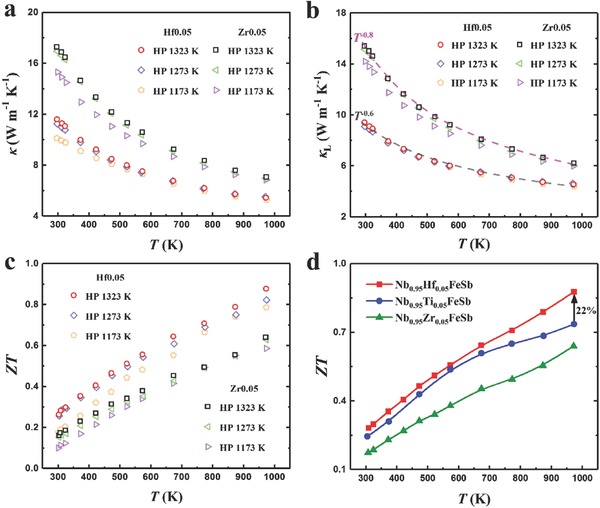
Temperature‐dependent a) total thermal conductivity, b) lattice thermal conductivity, and c) figure of merit *ZT* for Nb_0.95_Hf_0.05_FeSb and Nb_0.95_Zr_0.05_FeSb hot pressed at 1323, 1273, and 1173 K. d) Comparison of temperature‐dependent *ZT* among Nb_0.95_M_0.05_FeSb, together with the data of Ti doping.^33^

**Figure** **4**a shows the temperature‐dependent total thermal conductivity κ of Nb_0.95_Hf_0.05_FeSb and Nb_0.95_Zr_0.05_FeSb hot pressed at 1173, 1273, and 1323 K, which is dependent on either doping element or HP temperature. As known, κ includes the contributions of lattice κ_L_ and electronic κ_e_, where κ_e_ = *LσT* (*L* is the Lorenz number estimated by the SPB model),

(3) $$L\;=\;{\left(\frac{k_{B}}{e}\right)}^{2}\;\left[\frac{3F_{2}\left(\eta_{F}\right)}{F_{0}\left(\eta_{F}\right)}\;-\;{\left(\frac{2F_{1}\left(\eta_{F}\right)}{F_{0}\left(\eta_{F}\right)}\right)}^{2}\right]$$

The lower κ for the samples hot pressed at lower temperature is due to the reduced σ. By subtracting the κ_e_ from κ, the κ_L_ are presented in Figure 4b, which is slightly affected by HP temperature, suggesting little influence of the grain boundary scattering on the phonon transport. This experimental observation is consistent with our previous studies on Nb_0.95_Ti_0.05_FeSb, which is due to the dominant phonon mean free paths within the range of a few tens to a few hundreds of nanometers that are smaller than the grain size.^33^ However, the relatively large κ_L_ of Zr‐ and Ti‐doped samples almost offset the improvement of PF, but the Hf‐doped samples are noticeable for the significantly suppressed κ_L_. As Glassbrenner and Slack suggested, κ_L_ follows *T* ^−1^ law if only considering Umklapp phonon scattering process at temperature higher than the Debye temperature.^65^ Note that the κ_L_ of all samples shows *T^−k^* dependence with the exponent *k* of ≈0.8 for Zr doping and ≈0.6 for Hf doping, respectively. The reduced exponent *k* in Hf‐doped samples implies a strong point defect phonon scattering.^19^, ^20^ Hence the κ_L_ of Hf‐doped samples is significantly suppressed owing to the strong mass fluctuation and surrounding strain field fluctuation between Nb and Hf atoms (see details in the Supporting Information). Regardless of doping element, owing to improved PF and almost unchanged κ_L_ at higher HP temperature, *ZT* increased by simply increasing HP temperature, as shown in Figure 4c. More importantly, among the materials Nb_0.95_M_0.05_FeSb (M = Hf, Zr, Ti) with ultrahigh PF, Nb_0.95_Hf_0.05_FeSb hot pressed at 1323 K distinguishes itself for simultaneous optimization of PF and suppression of κ_L_, leading to a peak *ZT* of ≈0.9 at 973 K, which is 22% and 37% higher than those of Ti‐ and Zr‐doped samples shown in Figure 4d.

**Figure 5:**
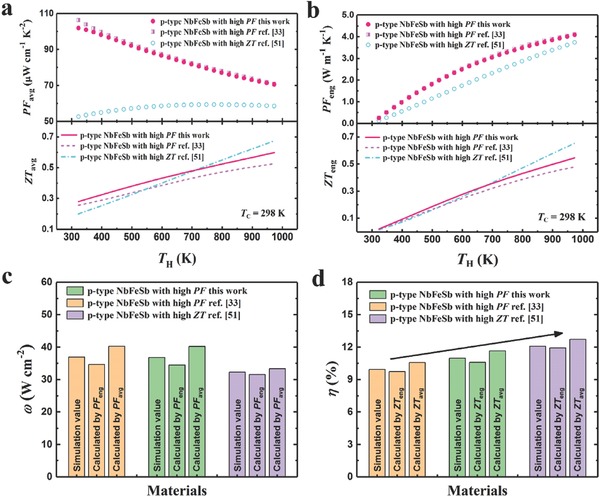
Comparison of a) average PF and average *ZT*, b) engineering PF and *ZT*, c) output power density, and d) efficiency between Nb_0.95_Hf_0.05_FeSb and other outstanding NbFeSb‐based materials.^33^, ^51^

As aforementioned, both the output power density ω and conversion efficiency η are crucial to power generation. In literature, average values (PF_avg_ and *ZT*_avg_) were used to calculate the ω and η, but they are not as reliable as the engineering values (PF_eng_ and *ZT*_eng_).^8^ **Figure** **5**a,b shows the detailed comparison of average values and engineering values, respectively, among Nb_0.95_Hf_0.05_FeSb (hot pressed at 1323 K) and other outstanding p‐type NbFeSb, e.g., Nb_0.95_Ti_0.05_FeSb with high PF over a wide range of temperature^33^ and Nb_0.86_Hf_0.14_FeSb with peak *ZT* of ≈1.5 at 1200 K.^51^ It is shown that Nb_0.95_Hf_0.05_FeSb has basically the same *T*_H_‐dependence (*T*_H_ and *T*_C_ are hot‐side and cold‐side temperature, respectively) of PF_avg_ and PF_eng_, but higher *ZT*_avg_ and *ZT*_eng_ over the whole temperature range compared with high‐PF material Nb_0.95_Ti_0.05_FeSb. For the high‐*ZT* material Nb_0.86_Hf_0.14_FeSb being concerned, it is very clear that both PF_avg_ and PF_eng_ of Nb_0.95_Hf_0.05_FeSb are much higher within the overall temperature range even though the *ZT*_avg_ and *ZT*_eng_ of Nb_0.86_Hf_0.14_FeSb are higher at above 700 K. By further investigation of thermoelectric performance among these p‐type HH compounds, the calculated ω based on PF_avg_ and PF_eng_ are shown in Figure 5c and η based on *ZT*_avg_ and *ZT*_eng_ shown in Figure 5d (*T*_C_ and *T*_H_ are 298 and 973 K, respectively). To demonstrate the adequacy of the calculated values, numerical simulations of ω and η based on the finite difference method are also presented (both the calculated ω and η based on engineering values have a smaller margin of error). Noticeably, compared with Nb_0.95_Ti_0.05_FeSb,^33^ the η of Nb_0.95_Hf_0.05_FeSb shows 11% enhancement, while the ω are almost the same. By comparison with Nb_0.86_Hf_0.14_FeSb,^51^ Nb_0.95_Hf_0.05_FeSb has a 14% higher in ω though η is 9.8% lower, which is very significant in the specific applications (e.g., unlimited heat supply or heat sink) where ω is more important.^31^ Thus, our Nb_0.95_Hf_0.05_FeSb is more suitable for power generation in case of high ω is required but also with decent η.

**Figure 6:**
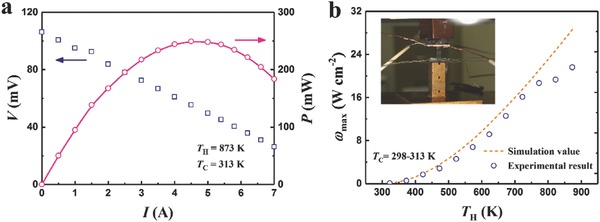
Measurement of output power density of Nb_0.95_Hf_0.05_FeSb. a) The measured voltage and power with varying applied electric current at *T*_C_ of ≈313 K and *T*_H_ of ≈873 K. b) *T*_H_‐dependent maximum output power density (normalized to the length of 2 mm), and the inset shows the experimental setup of the single‐leg device.

To corroborate the ultrahigh PF of Nb_0.95_Hf_0.05_FeSb, a single‐leg device was performed for output power density measurement in a homemade system^66^ (details are available in the Supporting Information). **Figure** **6**a shows the obtained voltage (*V*) and power (*P*) as a function of the applied electric current (*I*) at the *T*_C_ of ≈313 K and *T*_H_ of ≈873 K. Figure 6b shows the *T*_H_‐dependent maximum output power density (ω_max_) and the inset shows the experimental setup of the single‐leg device. Note that when *T*_H_ is above 773 K, the measured result deviates more from the simulation value, which was mainly due to the rise of *T*_C_ (the simulation assumes a fixed *T*_C_ of 298 K). In spite of that, a large ω_max_ of ≈21.6 W cm^−2^ was obtained at the *T*_C_ of ≈313 K and *T*_H_ of ≈873 K, demonstrating the excellent performance of Nb_0.95_Hf_0.05_FeSb in power generation.

## 3 Conclusion

In summary, by simultaneous optimization of carrier mobility and suppression of lattice thermal conductivity, a peak *ZT* of ≈0.9 at 973 K with ultrahigh PF over 100 µW cm^−1^ K^−2^ at room temperature was achieved in Nb_0.95_Hf_0.05_FeSb. In addition to peak values of *ZT* and PF, detailed studies of average values and engineering values demonstrate unique performance of Nb_0.95_Hf_0.05_FeSb for power generation in comparison with other outstanding NbFeSb‐based materials. As expected, a large output power density of ≈21.6 W cm^−2^ was achieved based on a single‐leg device of Nb_0.95_Hf_0.05_FeSb under a temperature difference of ≈560 K, showing the great potential for realizing high power thermoelectric generators.

## 4 Experimental Section

*Synthesis*: The samples with nominal compositions of Nb_0.95_Hf_0.05_FeSb and Nb_0.95_Zr_0.05_FeSb were prepared by arc melting, ball milling, and hot pressing process. The stoichiometrically weighed raw materials (Nb foils, 99.95%; Fe granules, 99.98%; Sb shots, 99.8%; Hf sponges, 99.9%; Zr sponges, 99.2%) were melted for several times in an Ar‐protected chamber to form homogeneous ingots. Subsequently, the ingots were loaded into the Ar‐filled stainless steel jar and ball milled for 3 h. The ball‐milled powders were finally consolidated into disks with diameter of ≈12.7 mm via direct‐current assisted hot pressing process. The hot pressing temperatures for each composition were at 1173, 1273, and 1323 K with holding for 2 min.

*Structure Characterization*: The phases of samples were characterized by an XRD instrument (PANalytical X'Pert Pro). Scanning electron microscope (LEO 1525) was applied to reveal the morphology of samples. The elemental mapping was performed by energy‐dispersive X‐ray spectroscopy (JEOL 6340F).

*Thermoelectric Measurement*: Thermal conductivity were calculated according to κ = *DC*_P_ρ, where *D* is the thermal diffusivity measured in a laser flash instrument (LFA457, Netzsch), *C*_P_ is the specific heat measured in a differential scanning calorimeter apparatus (DSC 404 C, Netzsch), and ρ is the mass density obtained by Archimedes method. Bar‐shaped samples cut from the disks, were performed for simultaneous measurement of Seebeck coefficient and electrical conductivity in a ZEM‐3 system (ULVAC). Carrier concentration (*n*_H_) at room temperature was measured in a physical properties measurement system (Quantum Design) and Hall mobility (*µ*_H_) were calculated based on σ = *n*_H_ *eµ*_H_, where *e* is the electronic charge. As shown in Table S1 (Supporting Information), the *n*_H_ results were quite close to the theoretical expectation by assuming one carrier from one doping atom (≈9.5 × 10^20^ cm^−3^), indicating that both Hf and Zr were very effective p‐type dopants in NbFeSb.

## Conflict of Interest

The authors declare no conflict of interest.

## Supporting information

Supplementary
