# Supplementary material for: Ultrahigh Power Factor in Thermoelectric System Nb0.95M0.05FeSb (M = Hf, Zr, and Ti)
Source: Adv Sci (Weinh). 2018 May 2;5(7):1800278. doi: 10.1002/advs.201800278 (PMC6051200; doi:10.1002/advs.201800278)
Supplement: Supplementary file 1 — Supplementary [file ADVS-5-1800278-s001.pdf]

Copyright WILEY-VCH Verlag GmbH & Co. KGaA, 69469 Weinheim, Germany, 2018.

ADVANCED SCIENCE Open Access

Supporting Information

for *Adv. Sci.,* DOI: 10.1002/advs.201800278

Ultrahigh Power Factor in Thermoelectric System $Nb_{0.95}M_{0.05}FeSb$ (M = Hf, Zr, and Ti)

Wuyang Ren, Hangtian Zhu, Qing Zhu, Udara Saparamadu, Ran He, Zihang Liu, Jun Mao, Chao Wang, Kornelius Nielsch, Zhiming Wang, and Zhifeng Ren**

Copyright WILEY-VCH Verlag GmbH & Co. KGaA, 69469 Weinheim, Germany, 2016.

Supporting Information

Ultrahigh Power Factor in Thermoelectric System $Nb_{0.95}M_{0.05}FeSb$ (M = Hf, Zr, and Ti)

Wuyang Ren, Hangtian Zhu, Qing Zhu, Udara Saparamadu, Ran He, Zihang Liu, Jun Mao, Chao Wang, Kornelius Nielsch, Zhiming Wang, and Zhifeng Ren**

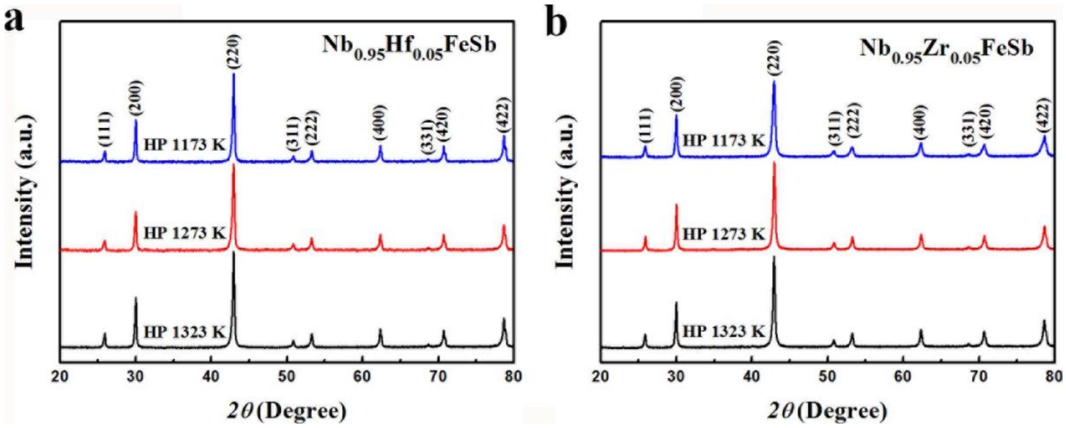

Figure S1. XRD spectra of $Nb_{0.95}Hf_{0.05}FeSb$ (a) and $Nb_{0.95}Zr_{0.05}FeSb$ (b) hot pressed at 1323 K, 1273 K, and 1173 K.

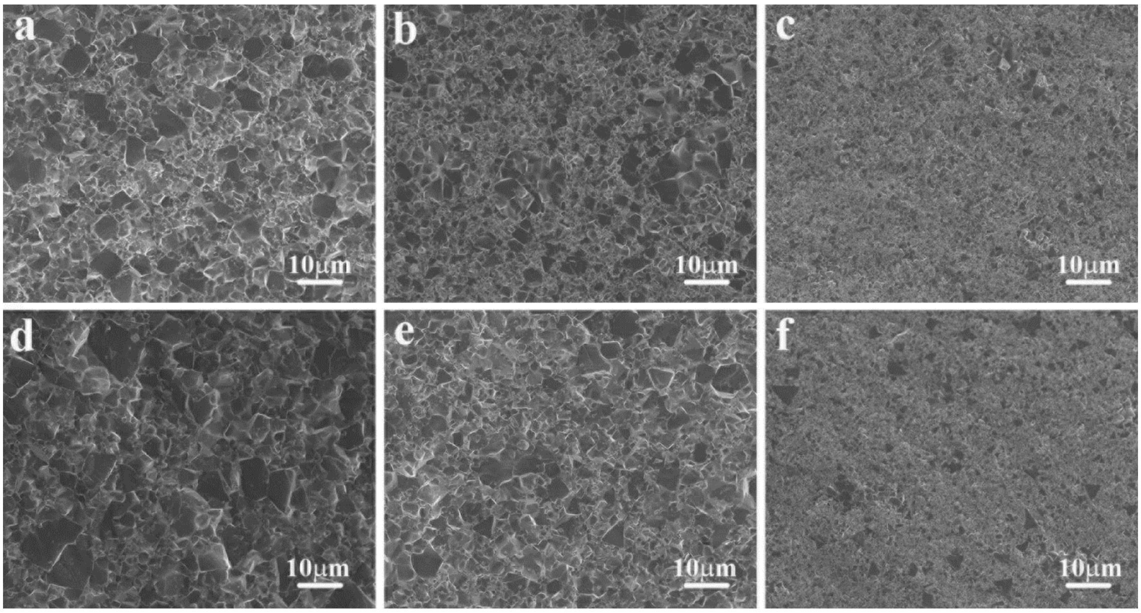

Figure S2. SEM images of $Nb_{0.95}Hf_{0.05}FeSb$ (a-c) and $Nb_{0.95}Zr_{0.05}FeSb$ (d-f) hot pressed at 1323 K, 1273 K, and 1173 K with low magnification.

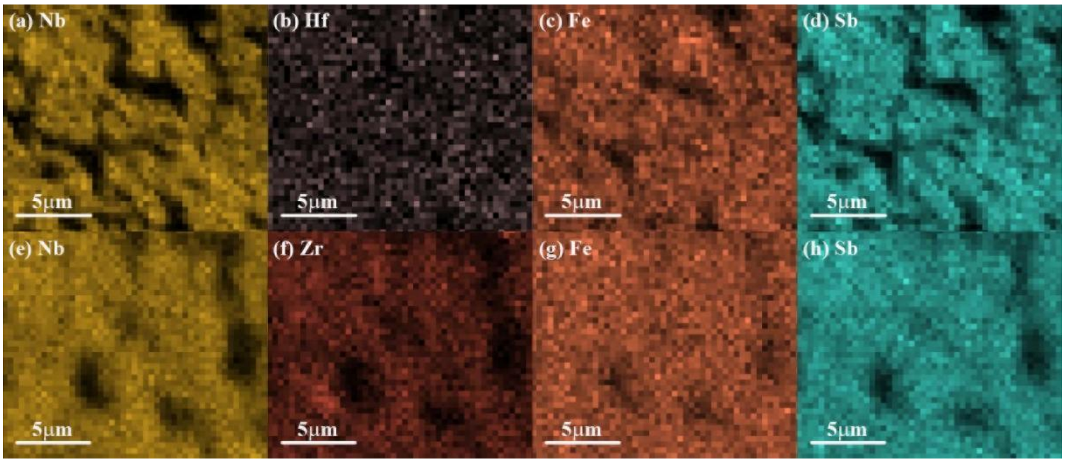

Figure S3. EDS elemental mapping of $Nb_{0.95}Hf_{0.05}FeSb$ (a-d) and $Nb_{0.95}Zr_{0.05}FeSb$ (e-h) hot pressed at 1323 K.

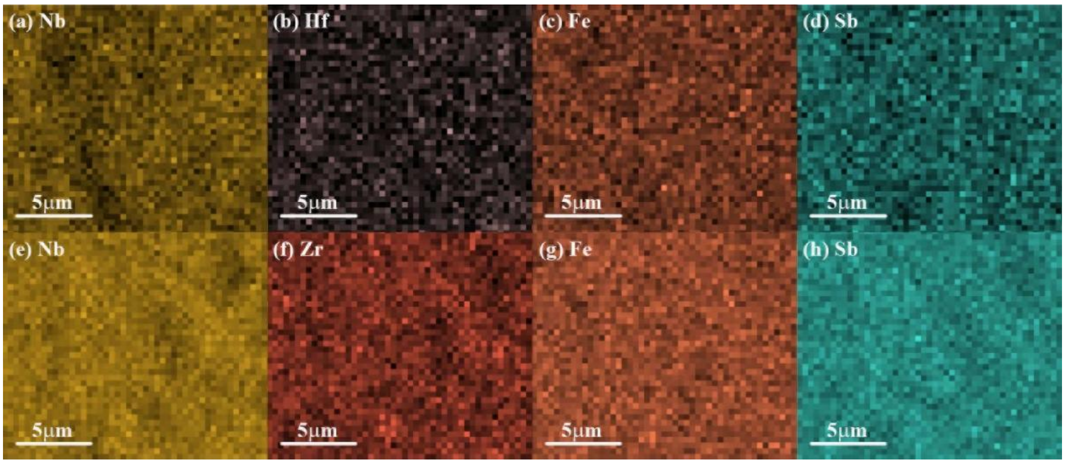

Figure S4. EDS elemental mapping of $Nb_{0.95}Hf_{0.05}FeSb$ (a-d) and $Nb_{0.95}Zr_{0.05}FeSb$ (e-h) hot pressed at 1173 K.

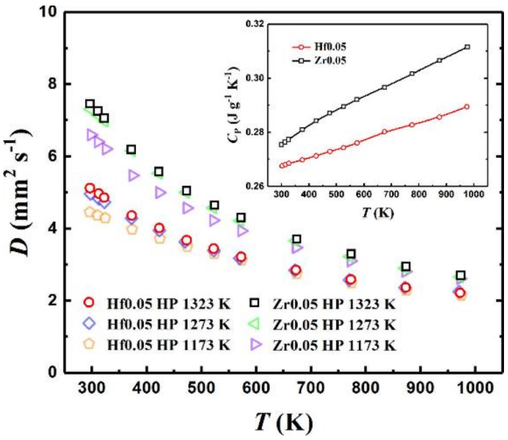

Figure S5. Temperature-dependent thermal diffusivity of $Nb_{0.95}Hf_{0.05}FeSb$ and $Nb_{0.95}Zr_{0.05}FeSb$ hot pressed at 1323 K, 1273 K, and 1173 K. And the inset shows the specific heat.

Table S1. Measured density, relative density and carrier concentration at 300 K of $Nb_{0.95}Hf_{0.05}FeSb$ and $Nb_{0.95}Zr_{0.05}FeSb$ hot pressed at 1323 K, 1273 K, and 1173 K

**Materials**	**HP temperature (K)**	**Measured density (g cm^{-3})**	**Relative density (%)**	**Carrier Concentration (×10^{20} cm^{-3})**
$Nb_{0.95}Hf_{0.05}FeSb$	1323	8.48	98.2	9.3
$Nb_{0.95}Hf_{0.05}FeSb$	1273	8.48	98.2	9.6
$Nb_{0.95}Hf_{0.05}FeSb$	1173	8.49	98.3	9.9
$Nb_{0.95}Zr_{0.05}FeSb$	1323	8.42	99.1	9.9
$Nb_{0.95}Zr_{0.05}FeSb$	1273	8.42	99.1	10.1
$Nb_{0.95}Zr_{0.05}FeSb$	1173	8.42	99.1	9.6

Calculation of mass fluctuation and strain field fluctuation.

According to Klemens model,[1] the relationship between κ_L of a crystal with disorder and that without disorder κ_{L0}, is

$$\frac{\kappa_L}{\kappa_{L0}} = \frac{\tan^{-1}(u)}{u} \quad (1)$$

$$u^2 = \frac{\pi^2 \theta_D V_0}{h v_s^2} \kappa_{L0} \Gamma \quad (2)$$

where u, θ_D, V_0, h, v_s, and Γ are the disorder scaling parameter, the Debye temperature, the average volume per atom, the Planck constant, the average lattice sound velocity, and the disorder scattering parameter, respectively. The disorder scattering parameter Γ is comprised of two parts, which derive from mass fluctuation Γ_M and strain field fluctuation Γ_S between the host atoms and the impurity atoms. For the ternary HH compounds, Γ is given by,[2]

$$\Gamma = \Gamma_M + \Gamma_S = \frac{1}{3}\left(\frac{\bar{M}}{\bar{\bar{M}}}\right) x(1-x)\left(\frac{M_1 - M_2}{\bar{M}}\right)^2 + \frac{1}{3}\left(\frac{\bar{M}}{\bar{\bar{M}}}\right) x(1-x)\varepsilon\left(\frac{r_1 - r_2}{\bar{r}}\right)^2 \quad (3)$$

$$\bar{M} = (1-x)M_1 + xM_2 \quad (4)$$

$$\bar{\bar{M}} = \frac{1}{3}(\bar{M} + M_3 + M_4) \quad (5)$$

$$\bar{r} = (1-x)r_1 + xr_2 \quad (6)$$

where x is the fractional concentration of doping element; M_1, M_2, M_3, and M_4 are the atomic mass of Nb, Hf (or Zr), Fe, and Sb, respectively; r_1 and r_2 are the atomic radius of Nb and Hf (or Zr), respectively; and ε is a phenomenological adjustable parameter. Here, the obtained Γ, Γ_M, and Γ_S are 1.82×10^{-2}, 1.30×10^{-2}, 0.52×10^{-2} by Hf doping, respectively; and for the similar atomic mass between Nb and Zr making Γ_M negligible, the Γ of Zr doped sample is mainly derived from the strain field fluctuation Γ_S which is 0.52×10^{-2}. Additionally, the values of ε (normally ranges from 10 to 100[2]) for Hf and Zr dopants are 56 and 49, respectively.

Calculation of average values, engineering values and the corresponding output power density and efficiency.

(i) Average *PF* and *ZT*, and the corresponding output power density and efficiency

$$PF_{avg} = \frac{1}{\Delta T}\int_{T_C}^{T_H} PF(T)dT \qquad (7)$$

$$ZT_{avg} = Z_{avg}T_{avg} = \frac{T_C+T_H}{2\Delta T}\int_{T_C}^{T_H} Z(T)dT \qquad (8)$$

$$\omega = \frac{(\Delta T)^2}{4L}PF_{avg} \qquad (9)$$

$$\eta = \frac{\Delta T}{T_H}\frac{\sqrt{1+ZT_{avg}}-1}{\sqrt{1+ZT_{avg}}+T_C/T_H} = \eta_C\frac{\sqrt{1+ZT_{avg}}-1}{\sqrt{1+ZT_{avg}}+T_C/T_H} \qquad (10)$$

(ii) Engineering *PF* and *ZT*, and the corresponding output power density and efficiency

$$PF_{eng} = \frac{\left(\int_{T_C}^{T_H} S(T)dT\right)^2}{\int_{T_C}^{T_H} \rho(T)dT} \qquad (11)$$

$$ZT_{eng} = \frac{PF_{eng}}{\int_{T_C}^{T_H} \kappa(T)dT}\Delta \mathrm{T} \qquad (12)$$

$$\omega = \frac{\Delta T}{4L}PF_{eng} \qquad (13)$$

$$\eta = \eta_C\frac{\sqrt{1+ZT_{eng}(\hat{\alpha}/\eta_C-1/2)}-1}{\hat{\alpha}\left(\sqrt{1+ZT_{eng}(\hat{\alpha}/\eta_C-1/2)}+1\right)-\eta_C} \qquad (14)$$

$$\hat{\alpha} = \frac{S(T_H)\Delta T}{\int_{T_C}^{T_H} S(T)dT} \qquad (15)$$

Here, L is the leg length of the thermoelectric material, and $\hat{\alpha}$ is a dimensionless intensity factor of the Thomson effect. Detailed definition and explanation are described in Ref. [3].

Measurement of output power density.

In this work the sample $Nb_{0.95}Hf_{0.05}FeSb$ was polished to size ~ 1.7×1.7 mm^2 in cross-section and ~ 5 mm in height. One side of thermoelectric leg soldered with copper plate was in contact with the cooling system to maintain a relatively stable temperature. Another side of thermoelectric leg brazed with copper plate was heated by contacting with a heater. By changing the power supply of heater, a series of temperature differences and voltages were produced through the thermoelectric leg. To measure the output power density, constant current was provided by another power source and voltage was measured by nanovoltmeter. K-type thermocouples were connected to copper plates to measure the temperature of both sides. The experiment was performed under high vacuum (below 10^{-6} mbar) to eliminate heat loss and oxidation of sample.

References

[1] P. G. Klemens, *Phys. Rev.* **1960**, *119*, 507.

[2] J. H. Yang, G. P. Meisner, L. D. Chen, *Appl. Phys. Lett.* **2004**, *85*, 1140.

[3] H. S. Kim, W. S. Liu, G. Chen, C. W. Chu, Z. F. Ren, *Proc. Natl. Acad. Sci. USA* **2015**, *112*, 8205.
